# Heterodimensional Structure Switching Multispectral Stealth and Multimedia Interaction Devices

**DOI:** 10.1002/advs.202302361

**Published:** 2023-07-10

**Authors:** Jin‐Cheng Shu, Mao‐Sheng Cao, Yan‐Lan Zhang, Wen‐Qiang Cao

**Affiliations:** ^1^ School of Materials Science and Engineering Beijing Institute of Technology Beijing 100081 China

**Keywords:** frequency‐selective antenna, heterodimensional structure, oxidative molecular layer deposition, multispectral electromagnetic wave stealth, strain imaging devices

## Abstract

Lightweight and flexible electronic materials with high energy attenuation hold an unassailable position in electromagnetic stealth and intelligent devices. Among them, emerging heterodimensional structure draws intensive attention in the frontiers of materials, chemistry, and electronics, owing to the unique electronic, magnetic, thermal, and optical properties. Herein, an intrinsic heterodimensional structure consisting of alternating assembly of 0D magnetic clusters and 2D conductive layers is developed, and its macroscopic electromagnetic properties are flexibly designed by customizing the number of oxidative molecular layer deposition (oMLD) cycles. This unique heterodimensional structure features highly ordered spatial distribution, with an achievement of electron‐dipole and magnetic–dielectric double synergies, which exhibits the high attenuation of electromagnetic energy (160) and substantial improvement of dielectric loss tangent (≈200%). It can respond to electromagnetic waves of different bands to achieve multispectral stealth, covering visible light, infrared radiation, and gigahertz wave. Importantly, two kinds of ingenious information interaction devices are constructed with heterodimensional structure. The hierarchical antennas allow precise targeting of operating bands (S‐ to Ku‐ bands) by oMLD cycles. The strain imaging device with high sensitivity opens a new horizon for visual interaction. This work provides a creative insight for developing advanced micro–nano materials and intelligent devices.

## Graphical abstract

A novel heterodimensional structure consisting of alternating assembly of 0D magnetic clusters and 2D conductive layers is developed, with high‐efficiency attenuation and conversion of electromagnetic energy, which can achieve multispectral stealth. Two kinds of information interaction devices are constructed, opening up a new horizon for wireless communication. This work provides a creative insight for developing advanced materials and intelligent devices.

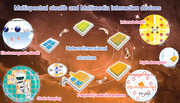

## 1 Introduction

The human society is undergoing a new round of scientific and technological revolution focusing on artificial intelligence, quantum communication, and metauniverse, which will completely overturn the traditional business model, industrial structure, and living habits, opening a new era of worldwide interconnection, digital economy, and intelligence of everything.^[^ ^1^, ^2^, ^3^ ^]^ Intelligent electromagnetic devices as important nodes in the internet of everything receive, process, and transmit various data and signals efficiently and accurately, achieving seamless connection between virtual network and real life.^[^ ^4^, ^5^, ^6^ ^]^ In recent years, along with the rapid development of functional materials and communication technology, the diversified intelligent electromagnetic devices such as nano–micro sensor and detector,^[^ ^7^ ^]^ far‐field imaging system,^[^ ^8^ ^]^ nanoantenna,^[^ ^9^ ^]^ electromagnetic wave absorber,^[^ ^10^ ^]^ and energy conversion cell^[^ ^11^ ^]^ emerge endlessly and are ardently sought after all over the world. Liu et al. developed a multifunctional Ti_3_C_2_T*_X_* MXene hydrogel, realizing sensitive pressure sensing and absorption‐dominated electromagnetic interference shielding.^[^ ^12^ ^]^ Kocabas and his partners proposed a novel multispectral graphene‐based electro‐optical surface, with reversible tunability from visible to microwave wavelengths.^[^ ^13^ ^]^ Continued advances in intelligent electromagnetic devices set off a research upsurge in the interdisciplinary field, laying a solid foundation for the progress of industry and technology.

Unfortunately, while electromagnetic devices are illuminating the progress of human civilization, the produced electromagnetic radiation also carries out a large‐scale bombardment on society, which exacerbates people's anxiety and panic.^[^ ^14^, ^15^ ^]^ The radiation of “big data” not only damages the internal system of the human body and causes cancer, but also leads to the abnormal growth and development of animals and plants, endangering the environment on which human life depends.^[^ ^16^ ^]^ In terms of production, mass electromagnetic radiation can interfere with the operation of equipment, cause data loss, and even result in explosions and fires.^[^ ^17^ ^]^ In addition, in the military realm, information warfare and radar countermeasure between countries are becoming increasingly fierce, which puts forward a new requirement for electromagnetic stealth.^[^ ^18^ ^]^ Therefore, the exploration and development of intelligent devices with electromagnetic protection and stealth are a hot research topic.

The development of intelligent electromagnetic devices highly depends on a continuous breakthrough in material preparation, structural design, and performance optimization.^[^ ^19^, ^20^, ^21^ ^]^ Micro–nano architecture features many intriguing physical properties, highlighting an important significance in the investigation of advanced functional materials and intelligent electromagnetic devices.^[^ ^22^ ^]^ In recent years, a novel heterodimensional structure has been developed, highly integrating the structural advantages of multi‐dimensional materials, which presents unique physical properties, such as electronic properties, magnetic properties, thermal properties, and others.^[^ ^23^ ^]^ Benefiting from this, the heterodimensional structure is setting off a new research upsurge in the intelligent devices and frontier fields.^[^ ^24^ ^]^ Regrettably, for heterodimensional structure, the precise fabrication with shape fidelity and design freedom is still very challenging, which will have an unpredictable effect on the performance of materials and devices.

Oxidative molecular layer deposition (oMLD), developed on the basis of atomic layer deposition (ALD), is a novel chemical vapor film preparation method based on ordered and surface auto‐saturation reactions.^[^ ^25^ ^]^ It allows the films of sub‐nanometer thickness to be deposited layer‐by‐layer on the complex surface of an object, thereby solving the problems of defects and uniformity nicely. In the preparation process, the alternate introduction of monomers and oxidants ensures the monolayer growth of molecular layer and precisely adjustable thickness.^[^ ^26^ ^]^ Meanwhile, the self‐limiting growth of the oMLD process ensures that the prepared films possess high density and good uniformity.^[^ ^27^ ^]^ Undoubtedly, the oMLD method can be applied to precise fabrication and controlled tailoring of heterodimensional structure, offering an infinite possibility in the development of advanced materials and intelligent devices. This is a meaningful subject and a rare opportunity.

In this work, a novel heterodimensional structure with periodic spatial distribution is controllably fabricated by confined recrystallization and oMLD approaches. For the first time, the intrinsic correlation between macroscopic electromagnetic properties and microscopic charge distribution is revealed by the sequencing of electromagnetic spectrum. The results confirm that the electron‐dipole and magnetic‐dielectric synergies dominate the electromagnetic loss and energy attenuation inside the heterodimensional structure, and the dissipation efficiency highly depends on frequency and oMLD cycle. The heterodimensional structure can serve as a broadband absorbing coating, realizing efficient absorption of electromagnetic wave and multispectral recognition. Importantly, two kinds of ingenious information interaction devices are constructed with the heterodimensional structure. The artificial magnetic conductor‐backed antennas feature high |S_11_| and satisfactory gains, and their operating bands can be flexibly switched from S‐ to Ku‐bands by customizing the oMLD cycles. Meanwhile, a novel strain imaging device is creatively developed, with integrated advantages including light weight, high sensitivity, tunable frequency, and wireless interaction. The fabricated heterodimensional structure shows devisable electromagnetic properties and high energy attenuation, injecting a new vitality into the development of advanced micro–nano architectures and intelligent electromagnetic devices.

## 2 Results and Discussion

**Figure 1:**
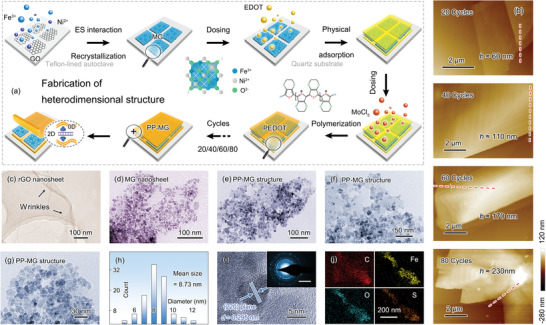
Schematic illustration of PP‐MG heterodimensional structure by confined recrystallization and oMLD process. a) Schematic synthesis process of PP‐MG heterodimensional structure. b) AFM images of heterodimensional structure with different PEDOT cycles. TEM images of c) rGO nanosheet, d) MG nanosheet, and e–g) PP‐MG heterodimensional structure. h) Size distribution of NiFe_2_O_4_ nanocrystals. i) High‐resolution TEM image and SAED pattern of heterodimensional structure. Scale bar in inset is 5 1/nm. j) Elemental mapping images of heterodimensional structure.

A novel heterodimensional structure is fabricated by confined recrystallization and oMLD approaches, which highly integrates the structural and componential advantages of 0D magnetic clusters and 2D rGO nanosheets/PEDOT layers (**Figure** **1**a). Impressively, the microstructure can be tailored precisely by adjusting the cycle numbers of oMLD process. The confined recrystallization is conducted via a hydrothermal practice. Graphene oxide (GO) nanosheets with oxygen‐containing functional groups can attract metal ions, Ni^2+^, and Fe^3+^ by electrostatic (ES) interaction for confined growth. Under high‐temperature and high‐pressure reaction environment, the metal ions at the confined sites hydrolyze, nucleate, and recrystallize into magnetic NiFe_2_O_4_ nanocrystals. The contest for oxygen possibly results in the in situ reduction of GO nanosheets. As the reaction proceeds, the magnetic graphene (MG) nanosheets are finally obtained. Notably, owing to the magnetic dipole–dipole attraction, the magnetic nanocrystals enable aggregation to form small clusters.^[^ ^28^ ^]^ The oMLD is conducted in a homemade ALD reactor (Figure S1, Supporting Information). The MG nanosheets dispersed into ethanol are dropped on a quartz substrate and then loaded into a chamber. The EDOT monomers and MoCl_5_ oxidants are heated to 60 °C and 80 °C, respectively, and the vapor phase precursors with reasonable vapor pressures are obtained. The extremely short dosing time of 7 s for EDOT and 10 s for MoCl_5_ is adopted to precisely control the deposition of PEDOT layer on the MG nanosheet surface. The 60 s N_2_ purge after dosing is performed to ensure the complete removal of residual precursors and produced byproducts (HCl). The resulting heterodimensional structures feature symmetrical distribution in vertical direction. They are designated as *x* PP‐MG, where *x* represents the cycle number of PEDOT oMLD process. It is worth mentioning that the formed PEDOT layer can stably adhere to the MG nanosheet surface because of the strong physical adsorption between them, such as van der Waals and hydrogen bonding.^[^ ^24^ ^]^

Figure S2, Supporting Information, demonstrates the oxidative polymerization of EDOT monomers and conductive conversion of PEDOT layers. First, the EDOT monomers form an oxidation state by a single electron oxidation, along with a reduction of MoCl_5_ to MoCl_4_. According to the electrical neutrality principle, the combination of the extra Cl^−^ with the oxidized monomers makes the entire system appear uncharged. Next, a recombination followed by thiophene rearomatization occurs between oxidized EDOT monomers to finally polymerize into neutral PEDOT, along with the formation of byproducts (HCl). Last, an oxidative conversion is carried out to obtain conductive PEDOT. Compared with traditional ALD and MLD, the oMLD features significant advantages in improving conductivity (*σ*) and dielectric response.

The PP‐MG heterodimensional structure can be clearly identified by atomic force microscopy (AFM) images, as shown in Figure S3, Supporting Information. The formed NiF_2_O_4_ small clusters are relatively evenly distributed over the rGO nanosheets. After depositing the PEDOT layers, the fragmented MG nanosheets are skillfully bridged, thereby achieving a significant increment in the plane size from a few microns to tens of microns. More importantly, the thicknesses of the PEDOT layers can be tailored precisely by controlling oMLD cycles. As shown in Figure 1b, when the number of oMLD cycles is set to 20, 40, 60, and 80 times, the thickness of the PEDOT layers reaches ≈60, ≈110, ≈170, and ≈230 nm, respectively.

The microstructure of PP‐MG heterodimensional structure at different preparation stages is further insighted by the transmission electron microscopy (TEM) images. The rGO nanosheets, featuring light weight and flexibility, can form many wrinkle structures (Figure 1c). After a simple hydrothermal process, the magnetic NiFe_2_O_4_ small clusters are confinedly implanted over the rGO nanosheets. It should be noted that the excessive precursor ion implantation will lead to the accumulation of the resulting NiFe_2_O_4_ nanocrystals and eventually cover the entire surface of the rGO nanosheets (Figure 1d). Figure 1e,f shows the microstructure of PP‐MG heterodimensional structure. Benefiting from the stable bond between rGO nanosheets and NiFe_2_O_4_ small clusters, the implanted small clusters still maintain a stable distribution after depositing the PEDOT layers.^[^ ^29^ ^]^ As shown in Figure 1g,h, the NiFe_2_O_4_ nanocrystals with relatively uniform size are spherical and mostly around 5–12 nm in diameter. The mean size is 8.73 nm. The lattice structure of NiFe_2_O_4_ nanocrystal is identified by high‐resolution TEM image (Figure 1i). The spacing of lattice fringe is 0.295 nm, which corresponds to (220) plane.^[^ ^30^ ^]^ The selected area electron diffraction (SAED) patterns of NiFe_2_O_4_ nanocrystals verify its cubic crystal structure (Inset in Figure 1I; Figure S4, Supporting Information). Figure 1j shows the element distribution of PP‐MG heterodimensional structure. The overlap of C, Fe, O, and S elements is further evidence of its ordered spatial distribution.

Based on the atomic‐scale observations and first principles calculations, the material basis depended on by dielectric response inside PP‐MG heterodimensional structure is revealed reasonably, as shown in Figures S5 and S6, Supporting Information. The GO nanosheets prepared by oxidation process have a wealth of intrinsic defects and foreign adatoms (Figure S5a,b, Supporting Information). The common intrinsic defects are sorted into three categories, including Stone‐Wales defects, vacancy defects, and large‐scale defects.^[^ ^31^, ^32^, ^33^ ^]^ The foreign adatoms are mainly indexed to oxygen‐containing functional groups, involving hydroxyl, carboxyl, and epoxy groups.^[^ ^34^ ^]^ After the reduction, the partial intrinsic defects and foreign adatoms are reserved for rGO nanosheets. Figure S5c, Supporting Information, shows the atomic‐scale phases of the Stone‐Wales defects, double‐vacancies, and epoxy groups inside rGO nanosheets (from left to right). These defects will cause lattice distortion and induce asymmetric charge distribution, which can be strongly supported by the calculated electron densities (Figure S5e, Supporting Information) and density of states (Figure S5f, Supporting Information).

After implanting NiFe_2_O_4_ small clusters, the rGO nanosheets are endowed with additional magnetic properties. Meanwhile, defects may be formed inside NiFe_2_O_4_ nanocrystals that will generate dipoles.^[^ ^35^, ^36^, ^37^, ^38^, ^39^ ^]^ Unfortunately, these magnetic clusters destroy the transport channels of charges on the rGO nanosheets, inevitably leading to a decline in the *σ* of the entire material system. To solve this problem, a controllable patching engineering based on oMLD approach is proposed for the first time. As shown in Figure S6a, Supporting Information, through the customization of oMLD cycles, the microcurrent network inside the heterodimensional structure is controllably reconstructed to achieve the precise design of *σ*. Notably, the implantation process of NiFe_2_O_4_ small clusters and the deposition process of PEDOT layers will increase the lattice distortion of rGO nanosheets, creating more defects inside, which can be identified through the calculated and experimental Raman spectra (Figures S5d and S6b,c, Supporting Information).^[^ ^40^ ^]^ In addition to defects, interface structure can also result in differential charge distribution. Figure S6d, Supporting Information, shows the high‐resolution TEM images of MG and PP‐MG, and the AFM images of PEDOT layers. It can be clearly observed that the abundant interfaces form among NiFe_2_O_4_ nanocrystals, NiFe_2_O_4_ nanocrystals and rGO nanosheets, rGO nanosheets and PEDOT layers, and others. Due to the difference in polarity or *σ* of rGO nanosheets, NiFe_2_O_4_ nanocrystals, and PEDOT layers, two sides of the interface possess different bound charge capacities under the electromagnetic field, thereby forming interface dipole.

**Figure 2:**
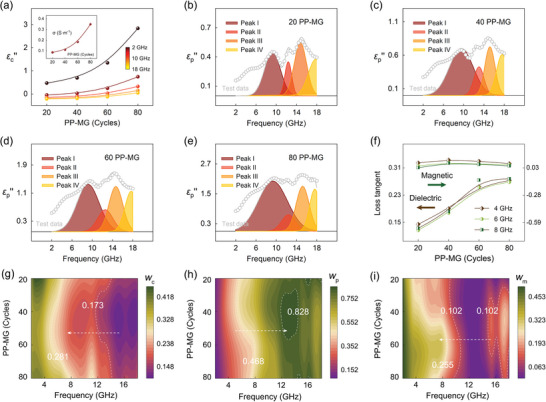
oMLD switched electromagnetic response and energy conversion. a) 2D plots of *ε*_c_" and *σ* versus PEDOT cycle. Frequency characteristics of *ε*_p_”” and the fitted relaxation peaks for heterodimensional structure with b) 20, c) 40, d) 60, and e) 80 PEDOT cycles. f) Dielectric and magnetic loss tangent of heterodimensional structure at 4, 6, and 8 GHz. Attenuation and conversion of electromagnetic energy driven by g) charge transport, h) dipole polarization, and i) magnetic response.

The response of charges and dipoles on the microstructure to electromagnetic wave is visually reflected in the macroscopic dielectric properties (Figure S7a, Supporting Information). The real permittivity (*ε′*) represents the storage capability of heterodimensional structure to energy, while the imaginary permittivity (*ε″*) indicates the inner dissipation capability of energy. According to Debye theory, the *ε″* depends on conduction loss (*ε*_c_ *″*) and relaxation loss (*ε*_p_ *″*), described as Equations (S1) and (S2), Supporting Information. **Figure** **2**a shows the evolution of the *ε*_c_ *″* of PP‐MG heterodimensional structure at different frequencies with the oMLD cycles, and the inset is the *σ* at different oMLD cycles. With the increase of oMLD cycles, the *σ* gradually raises, confirming the controllable patching of conductive PEDOT layers to the microcurrent network inside material system. Meanwhile, the *ε*_c_ *″* presents a distinct frequency and *σ* dependence. It decreases with the raised frequency but increases with the lifted *σ*, which is consistent with Debye theory. Figure 2b–e demonstrates the frequency characteristics of *ε*_p_ *″* of 20, 40, 60, and 80 PP‐MG heterodimensional structure, and four relaxation peaks are identified and fitted. Peaks I, II, and III are derived from MG nanosheets, and peak IV is caused by PEDOT layers.^[^ ^17^ ^]^

The magnetic properties of the heterodimensional structure are deeply dissected based on the real permeability (*µ'*) and imaginary permeability (*µ″*), as shown in Figure S7b,c, Supporting Information. The multiple resonance peaks are identified at the 2–18 GHz frequency band of the complex permeability, which is basically consistent with the standard magnetic resonance spectrum.^[^ ^41^, ^42^, ^43^, ^44^, ^45^ ^]^ Generally speaking, the peak located at frequency below 10 GHz is attributed to natural resonance, and exchange resonance is responsible for the origin of the peaks occurring at the frequency higher than 10 GHz. Among them, the appearance of the multiple exchange resonance peaks probably is caused by the difference in the size of domains. The magnetic eddy current is also an important source of the magnetic loss in the studied frequency band, which can heat the magnetic core to give rise to energy attenuation. The magnetic eddy current loss can be evaluated by magnetic eddy current coefficient (Equation (S3), Supporting Information). Once the coefficient is constant, the magnetic loss of PP‐MG heterodimensional structure just depends on the magnetic eddy current. However, due to the low *σ* of NiFe_2_O_4_ nanocrystals, the resulting eddy current loss is relatively feeble.

Figure 2f shows the dielectric and magnetic loss tangent of PP‐MG heterodimensional structure at different frequencies. As oMLD cycles are increased from 20 to 80 times, the dielectric loss tangent is improved by 200%, which means that the gradually perfected microcurrent network brings about stronger conduction loss and relaxation loss. Meanwhile, this result also demonstrates that the dielectric behavior of the heterodimensional structure can be precisely tuned through the customization of oMLD cycles. Besides, another noteworthy thing is that the oMLD, while tuning the dielectric loss, can also affect the magnetic loss tangent of the structure, which provides an important support for the achievement and controllable design of the magnetic–dielectric synergy.

The attenuation constant (*α*) is taken into account for evaluating the attenuation capability of PP‐MG heterodimensional structure to electromagnetic energy. As shown in Figure S7d, Supporting Information, the *α* raises with the increment of the frequency and oMLD cycles, with a maximum value up to 160. The high‐efficiency attenuation capability benefits from the advantage integration of PP‐MG heterodimensional structure in components and structure (Figure S7e, Supporting Information). First, the rational arrangement of dielectric and magnetic mediums achieves the multistage attenuation of electromagnetic energy. Second, the dual network structure constructed by rGO nanosheets and PEDOT layers provides abundant charges and transport channels, resulting in the improvement in *σ* and *ε*_c_ *″*. Third, the well‐designed spatial structures, including defects, functional groups, and interfaces, produce a wealth of dipoles, which cause a significant *ε*_p_" under alternating electromagnetic fields. Fourth, the large surface area of PP‐MG heterodimensional structure allows multiple scattering of electromagnetic wave, further facilitating the attenuation and conversion of electromagnetic energy.

The storage and conversion efficiency of electromagnetic energy inside PP‐MG heterodimensional structure is insighted in‐depth to further reveal its high attenuation behavior to the electromagnetic energy. Generally speaking, the electromagnetic energy can be divided into two parts after being absorbed, where one is stored inside the structure (*E*_s_) and the other is converted into waste heat energy (*E*_c_). Figure S7f, Supporting Information, shows the dependence of the ratio of *E*_c_ to *E*_s_ (*w*_r_) for four heterodimensional structures on frequency. The *w*_r_ gradually declines with the frequency, implying its poor adaptation at the high‐frequency band. Meanwhile, the *w*_r_ constantly rises with the increase in oMLD cycles, suggesting that the PEDOT layers can improve the conversion efficiency of electromagnetic energy, with a maximum value up to 33.2%. Figure S7g,h, Supporting Information, shows the storage (*w*_s_) and conversion efficiency (*w*_d_) of PP‐MG heterodimensional structure to the absorbed electromagnetic energy, and an opposite dependence of both of them on oMLD cycles is intuitively revealed.

The attenuation and conversion of electromagnetic energy are considered to depend on charge transport, dipole polarization, and magnetic response.^[^ ^11^ ^]^ Figure 2g–i demonstrates the conversion efficiency of electromagnetic energy contributed by the three (*w*_c_, *w*_p_, and *w*_m_). The charge transport dominates the energy conversion at the low‐frequency spectra, which is consistent with Debye theory. With the increment of frequency, the contribution of the charge transport gradually declines, and the importance of the dipole polarization is gradually highlighted. Notably, the maximum *w*_p_ value appears at ≈13 GHz rather than the highest frequency point because there are two exchange resonance peaks occurring at ≈15.5 and ≈17.5 GHz, bringing about a relatively strong magnetic loss. This phenomenon indicates that the dielectric and magnetic losses present a certain synergistic competition on the conversion of electromagnetic energy. At low‐frequency spectra, the natural resonance also makes an important contribution to energy conversion, and its efficiency is comparable to that of charge transport. In addition, due to the poor *σ* of the NiFe_2_O_4_ nanocrystals, the contribution of magnetic eddy current to the energy conversion is quite weak as to be negligible.

**Figure 3:**
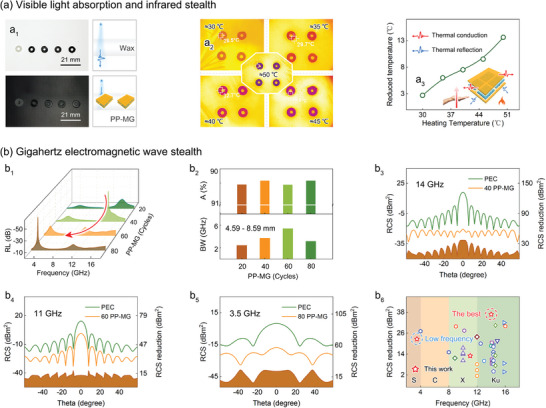
Multispectral electromagnetic wave stealth. a) Visible light absorption and infrared stealth. a_1_) Optical images of wax and PP‐MG/wax samples, and schematic diagram of visible light absorption. a_2_) Thermal infrared images at different heating temperatures. a_3_) 2D plot of reduced temperature versus heating temperature. b) Multi‐band electromagnetic wave stealth switched by oMLD cycles. b_1_) Migration tendency of the electromagnetic wave absorption peak driven by oMLD cycles. b_2_) Evaluation of absorption efficiency and effective BW. Far‐field electromagnetic wave absorption characteristics of heterodimensional structure in Ku‐, X‐, and S‐bands. RCS simulated curves and comparison of RCS reduction values: b_3_) 40 PP‐MG, b_4_) 60 PP‐MF, and b_5_) 80 PP‐MG. b_6_) Comparation of the RCS reduction values of PP‐MG heterodimensional structure with those of the previous works.

The PP‐MG heterodimensional structure enables response of electromagnetic waves of different bands, achieving multispectral stealth (**Figure** **3**). As shown in Figure 3a_1_, after the introduction of heterodimensional structure, the paraffin base sample changes from white translucent to black opaque, implying the effective absorption of heterodimensional structure to visible light, which can achieve the optical stealth in specific background. Meanwhile, the heterodimensional structure features good infrared stealth function. As shown in Figure 3a_2_, the color of the covered area turns purple in the field of view of infrared detection device, indicating the effective suppression of samples to thermal radiation. The maximum reduced temperature reaches 13.6 °C, which can be ascribed to the nice thermal conduction and thermal reflection caused by heterodimensional structure (Figure 3a_3_).

The PP‐MG heterodimensional structure can also absorb gigahertz electromagnetic wave, achieving precise electromagnetic stealth. More importantly, the far‐field electromagnetic wave absorption characteristics of the heterodimensional structure can be flexibly switched by customizing oMLD cycles, acquiring the selective response of the target bands. First of all, according to transmission line theory, the electromagnetic wave absorption performance of the heterodimensional structure is calculated (Figure S8a–d, Supporting Information), and the switched process based on the oMLD cycles is demonstrated. As shown in Figure 3b_1_, with the increase in the number of oMLD cycles, the strongest absorption peak shifts from high frequency to low frequency. The optimal absorption efficiency (*A*) exceeds 99.999% before and after transfer, which can be attributed to high‐efficiency electromagnetic loss and appropriate impedance matching (Figure 3b_2_; Figure S8e–h, Supporting Information). Generally speaking, when the impedance patching is close to 1, the first interface of the absorbers can effectively suppress reflection of incident electromagnetic wave to maximize absorption.^[^ ^46^ ^]^ Notably, dual absorption peaks appear as a transition state in the transfer process. At the moment, the absorption efficiency is significantly weakened, and the effective bandwidth (BW) (≤ −10 dB) is broadened to close to 6 GHz (Figure 3b_2_).

The radar cross section (RCS) is surveyed to investigate the far‐field electromagnetic wave absorption characteristics and stealth performance in the real environment.^[^ ^47^ ^]^ As shown in Figure S9a,b, Supporting Information, the PP‐MG heterodimensional structure as an absorption layer is coated on a metal plate (200 × 200 × 1 mm^3^), with an incident angle (theta angle) ranging from −60° to 60°. Figure 3b_3_; Figure S9c, Supporting Information, show the radar wave scattering signal of 40 PP‐MG structure as absorber layer. The RCS reduction values are more than 35 dBm^2^ at the theta angle of 0°, confirming the high‐efficiency absorption capacity of heterodimensional structure to radar wave (Figure S9d, Supporting Information). More meaningfully, the dominant response band can be flexibly switched from Ku‐ to S‐bands by increasing the number of oMLD cycles, as shown in Figure 3b_4_,_5_; Figure S9e–h, Supporting Information. Figure 3b_6_ compares the RCS reduction values (incident angle = 0°) of 40/60/80 heterodimensional structures with those of other works reported. As clearly observed, their performance is superior to those of most reported materials.

**Figure 4:**
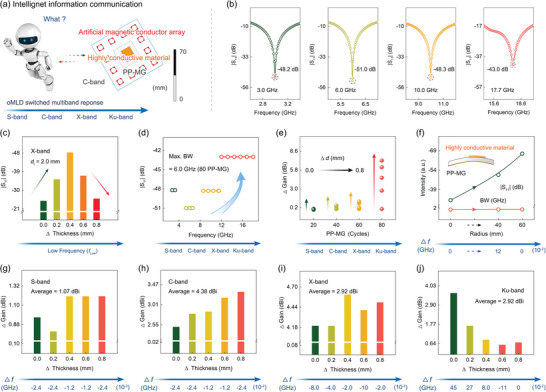
Intelligent information communication. a) oMLD switched intelligent information communication ranging from S‐ to Ku‐bands. b) |S_11_| of antennas composed of PEDOT patches and PP‐MG heterodimensional substrates. The resonance frequency can be switched from S‐ to Ku‐band by increasing PEDOT cycles from 20 to 80 times. c) Thickness‐dependance |S_11_| in X‐band. The peak frequency shifts toward low frequency with the increment of substrate thickness. d) The −10 dB impedance BWs corresponding to the optimal |S_11_| at S‐, C‐, X‐, and Ku‐bands. e) Increment of gains for antennas in the S‐, C‐, X‐, and Ku‐bands at different substrate thicknesses. f) The optimal |S_11_| and −10 dB impedance BW at different bending radii. The shift of the center frequency is always less than 0.12 GHz. The increased gains of the artificial magnetic conductor‐backed antennas and the shift of the center frequency at different thicknesses: g) S‐, h) C‐, i) X‐, and j) Ku‐bands.

The novel artificial magnetic conductor‐backed antennas are fabricated for the first time, composed of the artificial magnetic conductor array, wave‐transparent medium, lossy substrate constructed by PP‐MG heterodimensional structure, and highly conductive material patch, as shown in **Figure** **4**a; Figure S10, Supporting Information. The basic parameters of the antenna without artificial magnetic conductor are concerned first. Impressively, the communication bands can be flexibly switched from S‐ to Ku‐bands by customizing the electromagnetic properties of the substrate with oMLD cycles, as shown in Figure 4b. The |S_11_| values of the developed antennas are all less than −40 dB.

Figure 4c shows the dependence of the |S_11_| on the thickness of the substrate. With the increment of the thickness, the |S_11_| demonstrates an initial increase; and then, a subsequent decrease. The center frequency moves to low‐frequency band. Figure 4d shows the −10 dB impedance BWs of the antennas in the target bands. From S‐ to Ku‐bands, the BWs of the four antennas gradually increase and achieve complete coverage in Ku‐band (12–18 GHz), which is mainly driven by the increasingly enhanced electromagnetic loss for the substrates. Figure 4e shows the radiation gains of the four antennas at different substrate thicknesses, presenting a positively correlated dependence characteristic. With the increase in the substrate thickness, the gains gradually enlarge. Among them, the gain of the antenna in Ku‐band demonstrates the most sensitive response to the substrate thickness. When the thickness increases by 0.8 mm, the gain raises by 7.2 dBi. Furthermore, to explore the potential of the developed antenna as wearable devices, the bending simulation is carried out, with the radii of 0, 40, and 60 mm, respectively, as shown in Figure 4f. The |S_11_| values and −10 dB impedance BWs stably maintain ＜−30 dB and ≈3 GHz at different bending degrees, respectively, and the differences in the center frequency are within 0.12 GHz. It is well demonstrated that the antenna can be applied to flexible communication devices.

The artificial magnetic conductor arrays are introduced to enhance the radiation gain of the antennas for wider applications. As shown in Figure 4g–j, a significant improvement in the gains is achieved for the four antennas after assembling the artificial magnetic conductor arrays. The maximum average increment can reach 4.38 dBi in C‐band. The gain of an antenna mainly depends on efficiency and directivity. The assembly of artificial magnetic conductor array is exactly the improvement of the directivity by enhancing an in‐plane reflection effect, obtaining higher gain. Meanwhile, compared with other methods, the construction of array backplane can effectively avoid the second adjustment of antenna, thereby saving time and money cost.

**Figure 5:**
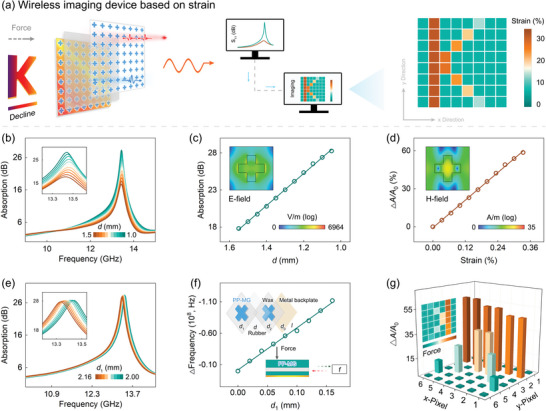
A novel wireless strain imaging device constructed by heterodimensional structure. a) Schematic diagram of wireless imaging based on strain. b) Frequency‐dependance absorption at different *d*. c) Fitted *d*‐response curve (*r* ^2^ = 0.9986). d) Fitted pressure‐response curve (*r* ^2^ = 0.9986). Insets in (c,d) are spatial variation of *E*‐field and *H*‐field, respectively. e) Frequency‐dependance absorption at different *d*_1_. f) Fitted *d*_1_‐response curve (*r* ^2^ = 0.9943). Insets in (f) are basic pixel of imaging device and capacitor‐like structure, respectively. g) Wireless image results based on different pressures.

The PP‐MG heterodimensional structure exhibits excellent electromagnetic response. Based on this, a creative wireless imaging device is constructed, which can resolve the electromagnetic signal that responds to pressure, achieving visualization of the acting object, as shown in **Figure** **5**a. The strain imaging devices consist of periodically arranged pixels, where capacitor‐like structure is constructed by patterned PP‐MG layers, elastic rubber, and metal backplate.

The novel imaging device is very sensitive to pressure. Among them, the pressure change is visually reflected in the strain of the rubber interlayer. When touching an object, the device is compressed to cause the decrease in the thickness of rubber interlayer. At the moment, the coupling effect between the two patterned PP‐MG layers enhances, enabling more efficient absorption and attenuation of incident electromagnetic wave (Figure 5b). A linear change of absorption peak value with thickness of rubber layer (*d*) can be fitted well (*r* ^2^ = 0.9986), with a sensitivity as high as 21585 dB m^−1^ (Figure 5c). Figure 5d further shows the linear relation of peak change (*A*/*A*_0_) with strain by *A*/*A*_0_ = 1.818 *d*/*d*_0_, where *d*_0_ is the thickness of rubber layer before compression. Insets in Figure 5c,d present spatial variation of electric field (E‐field) and magnetic field (H‐field) (*f* = 13.1 GHz), respectively.

More importantly, the peak frequency can be adjusted based on a non‐crosstalk mode by changing the thickness of the first patterned PP‐MG layer (*d*_1_), as shown in Figure 5e and the inset in Figure 5f. With the increase of *d*_1_, the absorption peak gradually shifts red. A linear evolution of peak frequency with *d*_1_ is demonstrated by ∆*f* (Hz) = −6.71 × 10^11^ ∆*d*_1_ (m), as shown in Figure 5f. Apparently, the strain imaging device with linear pressure sensing and non‐crosstalk frequency dependance is capable of achieving high‐precision wireless imaging (Figure 5g). The constructed information interaction devices are expected to be applied to artificial intelligence, wireless communications, aerospace, and other fields, which offers a brand new horizon for the development of intelligent devices.

## 3 Conclusion

A novel PP‐MG heterodimensional structure consisting of alternating assembly of 0D magnetic clusters and 2D conductive layers is controllably fabricated with confined recrystallization and oMLD process, and the precise design of macroscopic electromagnetic properties is achieved by customizing the number of oMLD cycles. The heterodimensional structure features symmetrical distribution in vertical direction, with an achievement of electron‐dipole and magnetic‐dielectric double synergies, which can dominate efficient attenuation and conversion of electromagnetic energy. Importantly, the developed heterodimensional structure enables response of electromagnetic waves of different bands to achieve multispectral stealth, including visible light, infrared radiation, and gigahertz wave. More impressively, two kinds of ingenious information interaction devices are constructed with PP‐MG heterodimensional structure. The artificial magnetic conductor‐backed antennas allow efficient reception and transmission of information, and their operating bands can be flexibly switched from S‐ to Ku‐bands by customizing the oMLD cycles. Meanwhile, a novel wireless imaging device based on strain is constructed, with a sensitivity up to 21 585 dB m^−1^, refreshing a cognizance to visual interaction. Undoubtedly, an important breakthrough has been made in material cognition, electromagnetic properties, and intelligent device, which offers an infinite possibility for the development of advanced materials and intelligent devices.

## Conflict of Interest

The authors declare no conflict of interest.

## Supporting information

Supporting Information

## Data Availability

The data that support the findings of this study are available from the corresponding author upon reasonable request.
