# Supplementary material for: Heterodimensional Structure Switching Multispectral Stealth and Multimedia Interaction Devices
Source: Adv Sci (Weinh). 2023 Jul 10;10(26):2302361. doi: 10.1002/advs.202302361 (PMC10502863; doi:10.1002/advs.202302361)
Supplement: Supplementary file 1 — Supporting Information [file ADVS-10-2302361-s001.pdf]

ADVANCED SCIENCE
Open Access

Supporting Information

for *Adv. Sci.*, DOI 10.1002/advs.202302361

Heterodimensional Structure Switching Multispectral Stealth and Multimedia Interaction Devices

Jin-Cheng Shu, Mao-Sheng Cao, Yan-Lan Zhang and Wen-Qiang Cao**

Supporting Information

Heterodimensional Structure Switching Multispectral Stealth and Multimedia Interaction Devices

Jin-Cheng Shu, Mao-Sheng Cao,* Yan-Lan Zhang, Wen-Qiang, Cao*

Dr. J.-C. Shu, Prof. M.-S. Cao, Dr. Y.-L. Zhang, Dr. W.-Q. Cao

[1]School of Materials Science and Engineering, Beijing Institute of Technology, Beijing 100081, China

E-mail: caomaosheng@bit.edu.cn; wenqiang_cao@sina.com;

J.-C.S. and M.-S.C. contributed equally to this work.

Experimental Section

Fabrication of PP-MG Heterodimensional Structure

Graphene oxide (GO) nanosheets were prepared by a modified Hummers' method.[1] Magnetic graphene (MG) was prepared by a facile hydrothermal method. Typically, 0.058 g of $Ni(NO_3)_2{\cdot}6H_2O$ and 0.162 g of $Fe(NO_3)_3{\cdot}9H_2O$ were dispersed evenly into GO suspension (0.7 $mg{\cdot}mL^{-1}$). $NH_3{\cdot}H_2O$ was used to adjust the pH value to 10. The obtained solution was transferred to 50 mL of Teflon-lined autoclave and maintained at 180 °C (24 h). Finally, the black magnetic powder was obtained by washing, drying, and grinding. PP-MG heterodimensional structure was fabricated by oxidative molecular layer deposition (oMLD), which was realized in a homemade atomic layer deposition (ALD) reactor. Typically, the MG nanosheets dispersed into ethanol were dropped on a quartz substrate. After being dried in air, the quartz substrate was transferred to ALD reactor. The PEDOT was deposited by sequential exposure of EDOT monomers and $MoCl_5$ oxidants at 115 °C. A complete cycle includes an exposure of EDOT monomer (7 s) followed by a N_2 purge (60 s) and an exposure of $MoCl_5$ oxidant (10 s) followed by a N_2 purge (60 s). The PEDOT cycles were repeated 20, 40, 60, 80 times, and the resulting products were denoted as 20 PP-MG, 40 PP-MG, 60 PP-MG, and 80 PP-MG, respectively.

Characterization and Simulation

The microstructure and elemental composition of the resultant PP-MG heterodimensional structure were recorded by transmission electron microscopy (TEM) and energy-dispersive X-Ray spectroscopy (EDX). The cross section of PP-MG structure was imaged by atomic force microscopy (AFM) (Bruker, Dimension FastScan). Raman spectra was obtained by Raman spectrometer (Renishaw, inVia, 514 nm). The complex permittivity and complex permeability (2-18 GHz) were measured by vector network analyzer (Anritsu, 37269D). The first principles calculation was performed by DMol3 module. The electromagnetic field response characteristic was investigated by CST Microwave Studio and High Frequency Structure Simulator.

Calculation of ε_c'', ε_p'', w_r, w_s, w_d, w_c, w_p, w_m, and σ_{RCS}

The contribution of conduction (ε_c'') and relaxation (ε_p'') to dielectric loss are defined as follow,

$$\varepsilon_c'' = \frac{\sigma}{\omega\varepsilon_0} \tag{1}$$

$$\varepsilon_p'' = \frac{\varepsilon_s - \varepsilon_\infty}{1 + \omega^2\tau^2}\omega\tau \tag{2}$$

where σ is the leakage conductivity. ε_0 is the vacuum permittivity. ω is the angular frequency ($\omega = 2\pi f$). τ is the relaxation time. ε_s and ε_∞ represent the static permittivity and the relative dielectric permittivity at high frequency limit (optical permittivity), respectively.

The magnetic eddy current coefficient is estimated by,

$$\mu''(\mu')^{-2} f^{-1} = 2\pi\mu_0\sigma d^2/3 \tag{3}$$

where μ_0 is the vacuum permeability.

The ratio of converted electromagnetic energy to stored electromagnetic energy (w_r) is calculated based on complex permittivity (ε' and ε'') and complex permeability (μ' and μ''),

$$w_r = \frac{\varepsilon''\varepsilon_0 E_0^2 + \mu''\mu_0 H_0^2}{\varepsilon'\varepsilon_0 E_0^2 + \mu'\mu_0 H_0^2} \tag{4}$$

The electromagnetic energy storage efficiency (w_s) and conversion efficiency (w_d),

$$w_s = \frac{\varepsilon'\varepsilon_0 E_0^2 + \mu'\mu_0 H_0^2}{\varepsilon'\varepsilon_0 E_0^2 + \varepsilon''\varepsilon_0 E_0^2 + \mu''\mu_0 H_0^2 + \mu'\mu_0 H_0^2} \tag{5}$$

$$w_d = \frac{\varepsilon''\varepsilon_0 E_0^2 + \mu''\mu_0 H_0^2}{\varepsilon'\varepsilon_0 E_0^2 + \varepsilon''\varepsilon_0 E_0^2 + \mu''\mu_0 H_0^2 + \mu'\mu_0 H_0^2} \tag{6}$$

where E_0 and H_0 represent the electric field intensity amplitude and the magnetic field intensity amplitude of EM wave, respectively.

Attenuation and conversion of electromagnetic energy driven by charge transport (w_c), dipole polarization (w_p), and magnetic response (w_m),

$$w_c = \frac{\varepsilon_c''\varepsilon_0 E_0^2}{\varepsilon''\varepsilon_0 E_0^2 + \mu''\mu_0 H_0^2} \tag{7}$$

$$w_p = \frac{\varepsilon_p''\varepsilon_0 E_0^2}{\varepsilon''\varepsilon_0 E_0^2 + \mu''\mu_0 H_0^2} \tag{8}$$

$$w_m = \frac{\mu''\mu_0 H_0^2}{\varepsilon''\varepsilon_0 E_0^2 + \mu''\mu_0 H_0^2} \tag{9}$$

The radar cross section value is defined as,

σ_{RCS} (dB m^2) = $10\log\left(\frac{4\pi S}{\lambda^2}\left|\frac{E_S}{E_i}\right|\right)^2$ (10)

where S and λ represent the area of the simulation model and wavelength of incident wave, respectively. E_S and E_i are the electric field intensity of scattered wave and incident wave, respectively.

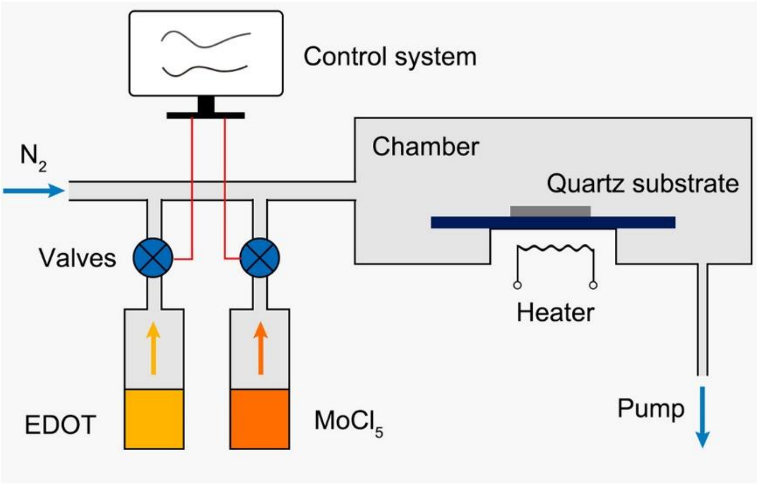

Figure S1. Schematic diagram of experimental set-up.

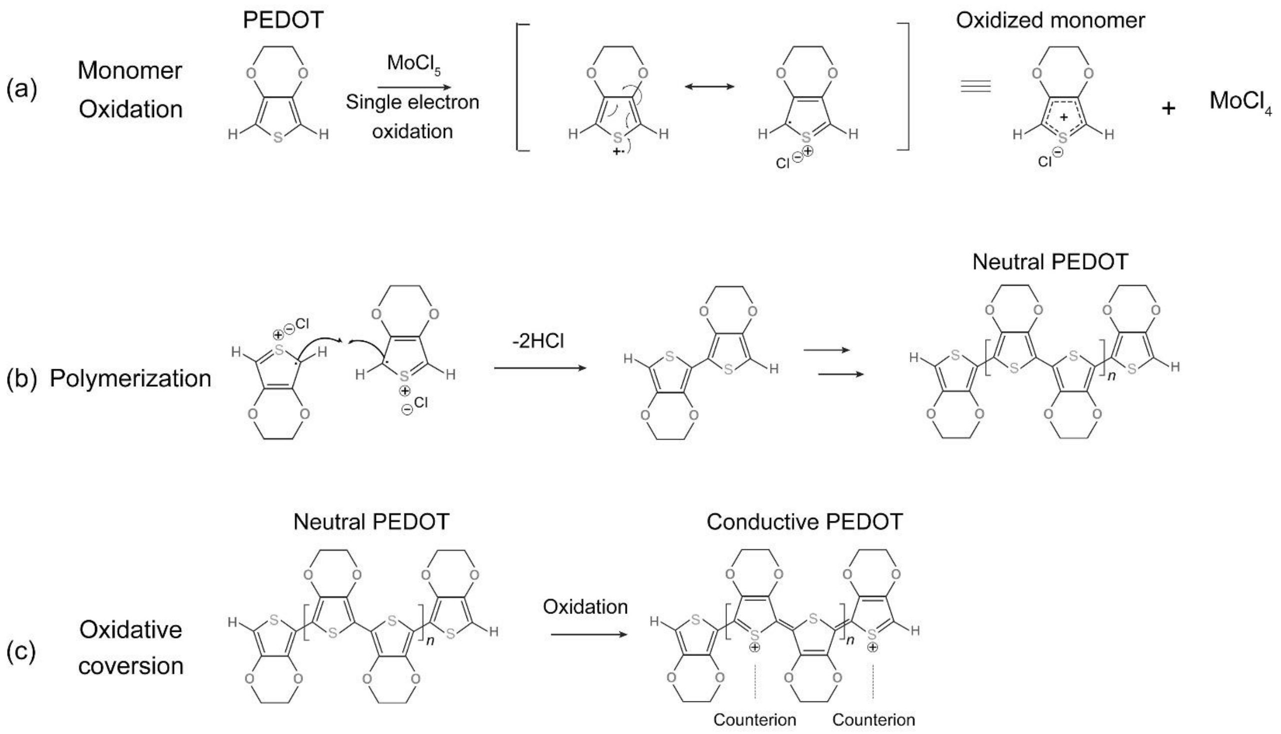

Figure S2. Oxidative polymerization of EDOT and conductive conversion of PEDOT.

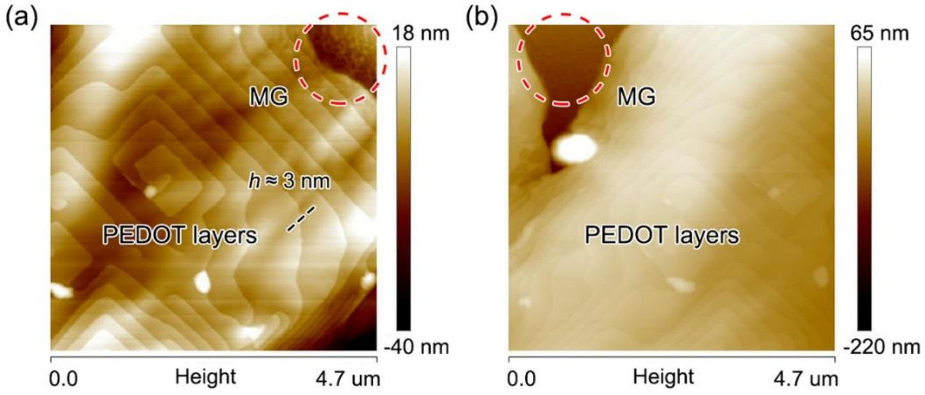

Figure S3. AFM images of PP-MG heterodimensional structure.

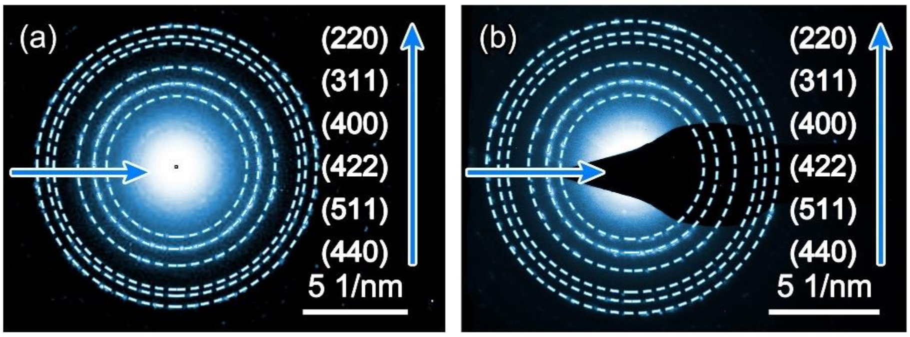

Figure S4. Selected area electron diffraction patterns of (a) MG nanosheets and (b) PP-MG heterodimensional structure.

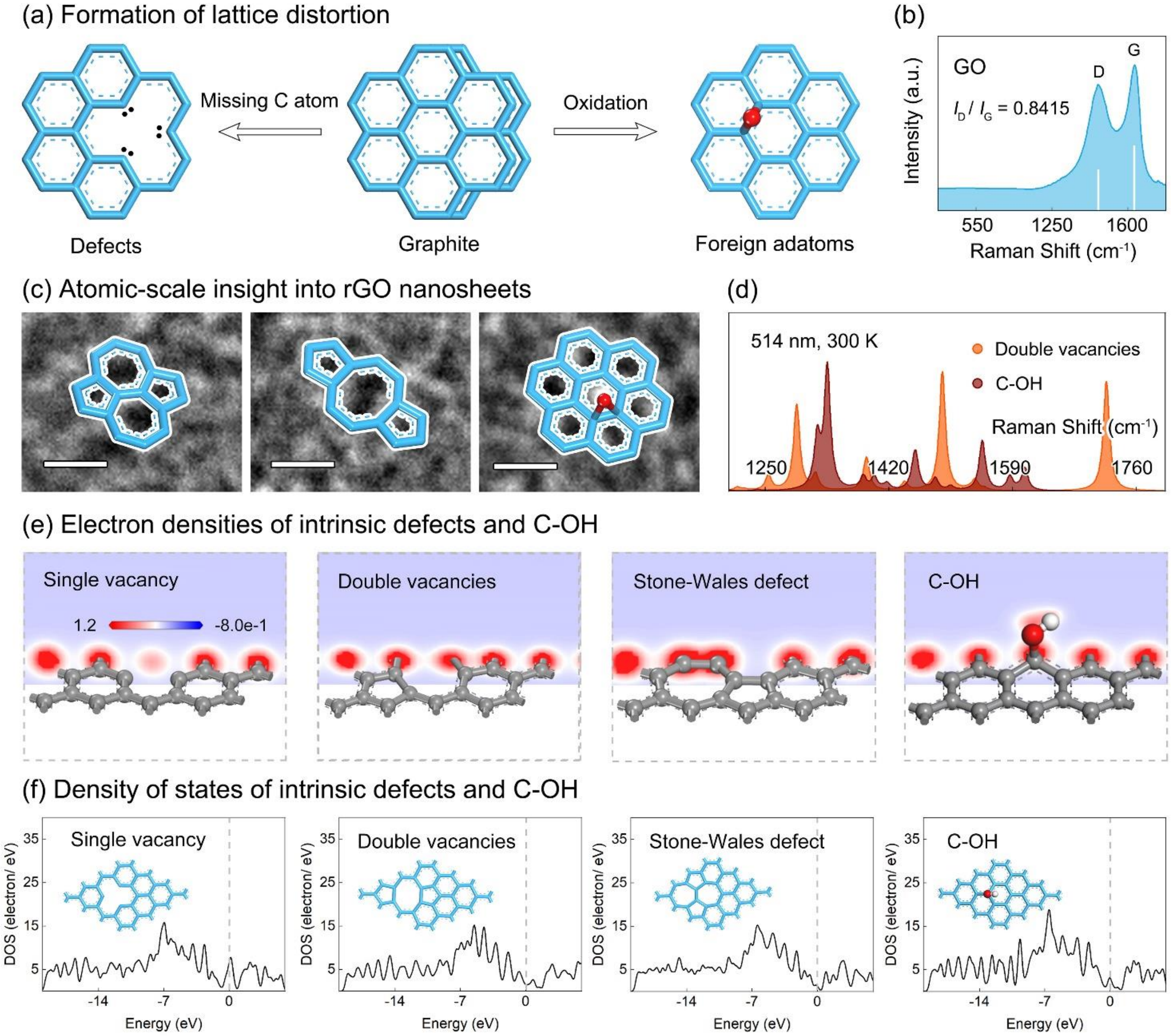

Figure S5. Atomic-scale insight into rGO nanosheets and first-principles calculations. a) Formation of intrinsic defects and foreign adatoms. b) Raman spectrum of GO nanosheets. c) Atomic-scale phase of Stone-Wales defect, double vacancies, and C-OH. Scale bars are 0.5 nm. d) Calculated Raman spectra of double vacancies and C-OH. e) Electron densities of intrinsic defects and C-OH. f) Density of states of intrinsic defects and C-OH.

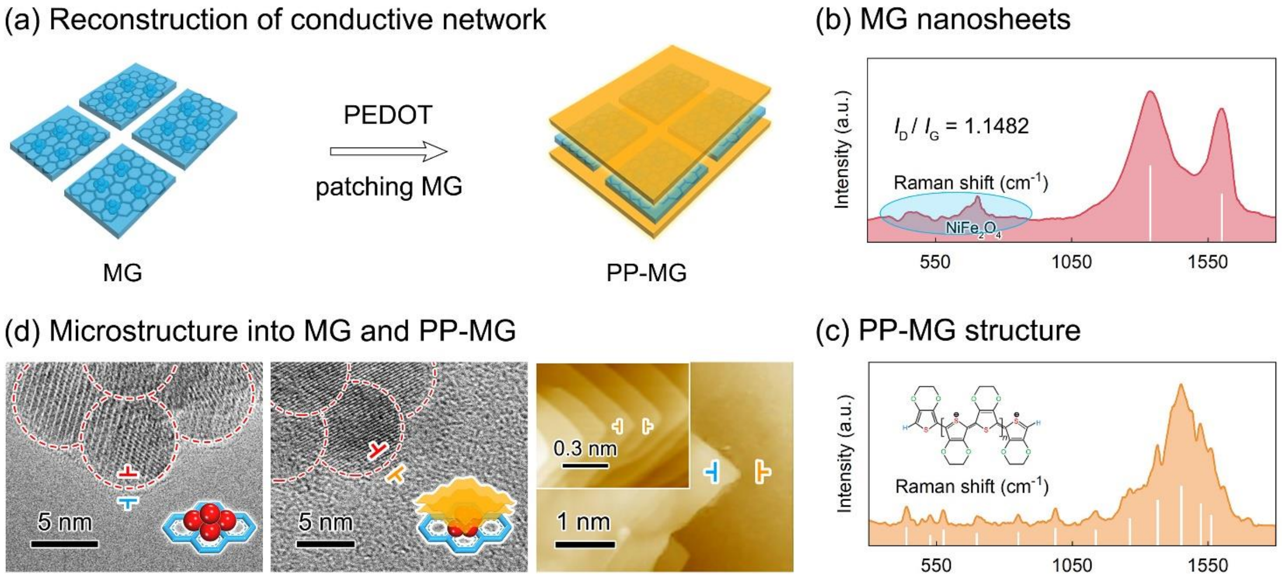

Figure S6. a) Reconstruction of conductive network. Raman spectra of (b) MG nanosheets and (c) PP-MG heterodimensional structure. The I_D/I_G value of PP-MG heterodimensional structure cannot be accurately evaluated due to the interference of PEDOT peaks. d) Microstructure of MG nanosheets and PP-MG heterodimensional structure.

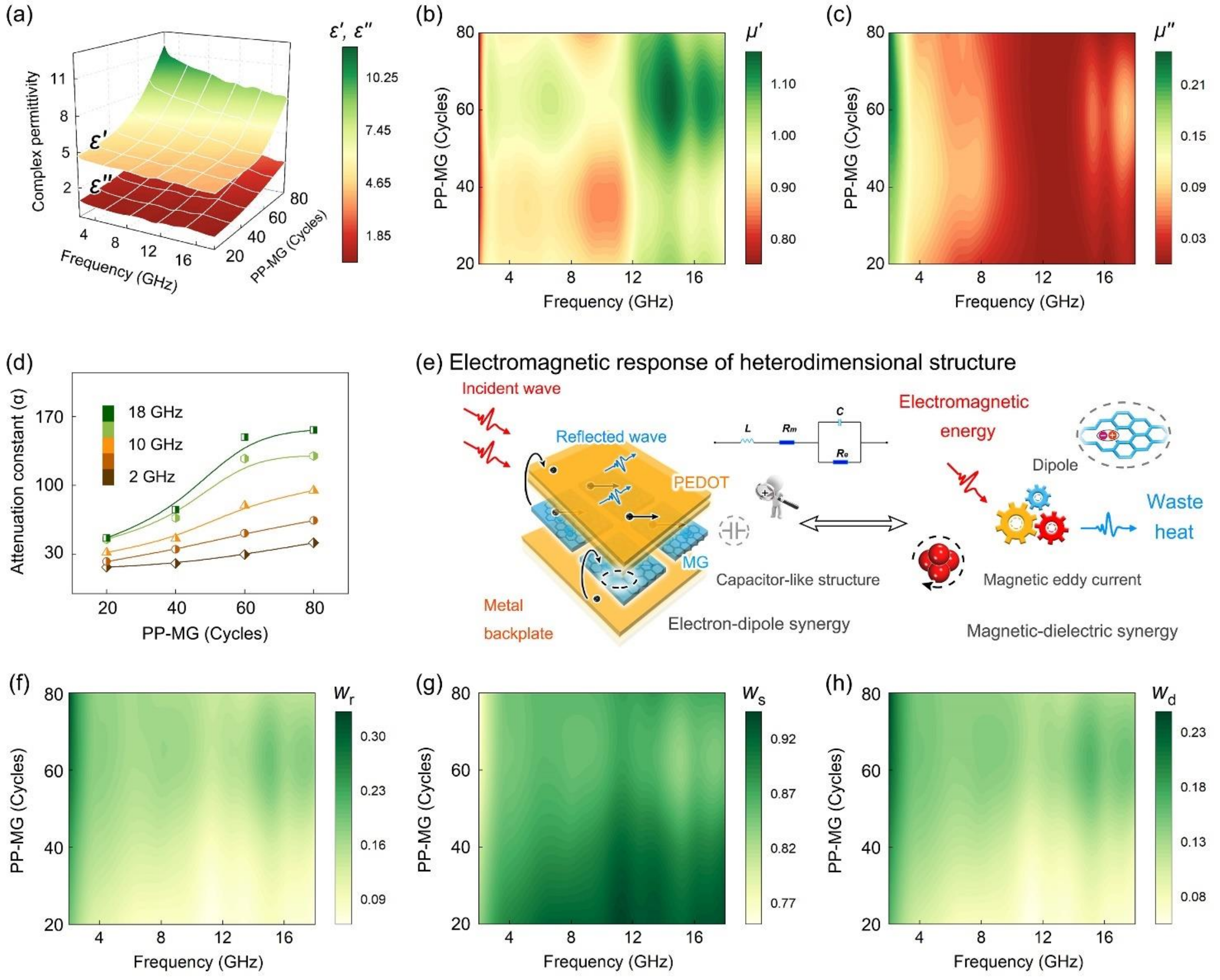

Figure S7. a) 3D plots of complex permittivity versus frequency and oMLD cycle. 3D plots of (b) real permeability and (c) imaginary permeability versus frequency and oMLD cycle. d) 2D plots of attenuation constant (α) versus PEDOT cycle at different frequencies. e) Electromagnetic response of heterodimensional structure, including charge transport, dipole relaxation, magnetic eddy current, and magnetic-dielectric synergy. Inset is equivalent circuit model. f) The ratio of converted electromagnetic energy to stored electromagnetic energy inside PP-MG heterodimensional structure (w_r). g) The electromagnetic energy storage efficiency inside PP-MG heterodimensional structure (w_s). h) The electromagnetic energy conversion efficiency inside PP-MG heterodimensional structure (w_d).

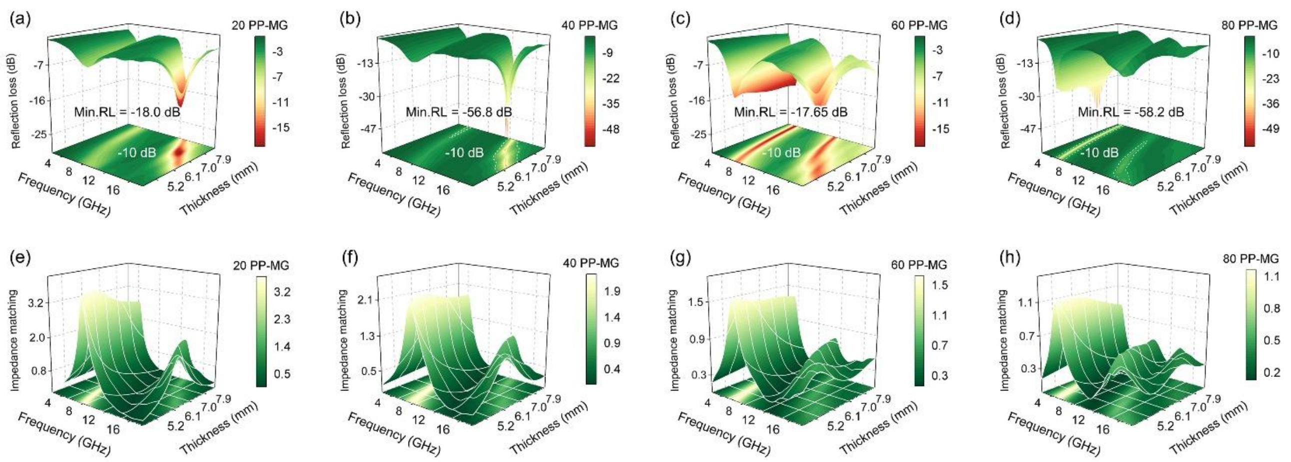

Figure S8. Multiband electromagnetic wave absorption performance of (a) 20 PP-MG, (b) 40 PP-MG, (c) 60 PP-MG, and (d) 80 PP-MG heterodimensional structure. Impedance matching of (e) 20 PP-MG, (f) 40 PP-MG, (g) 60 PP-MG, and (h) 80 PP-MG.

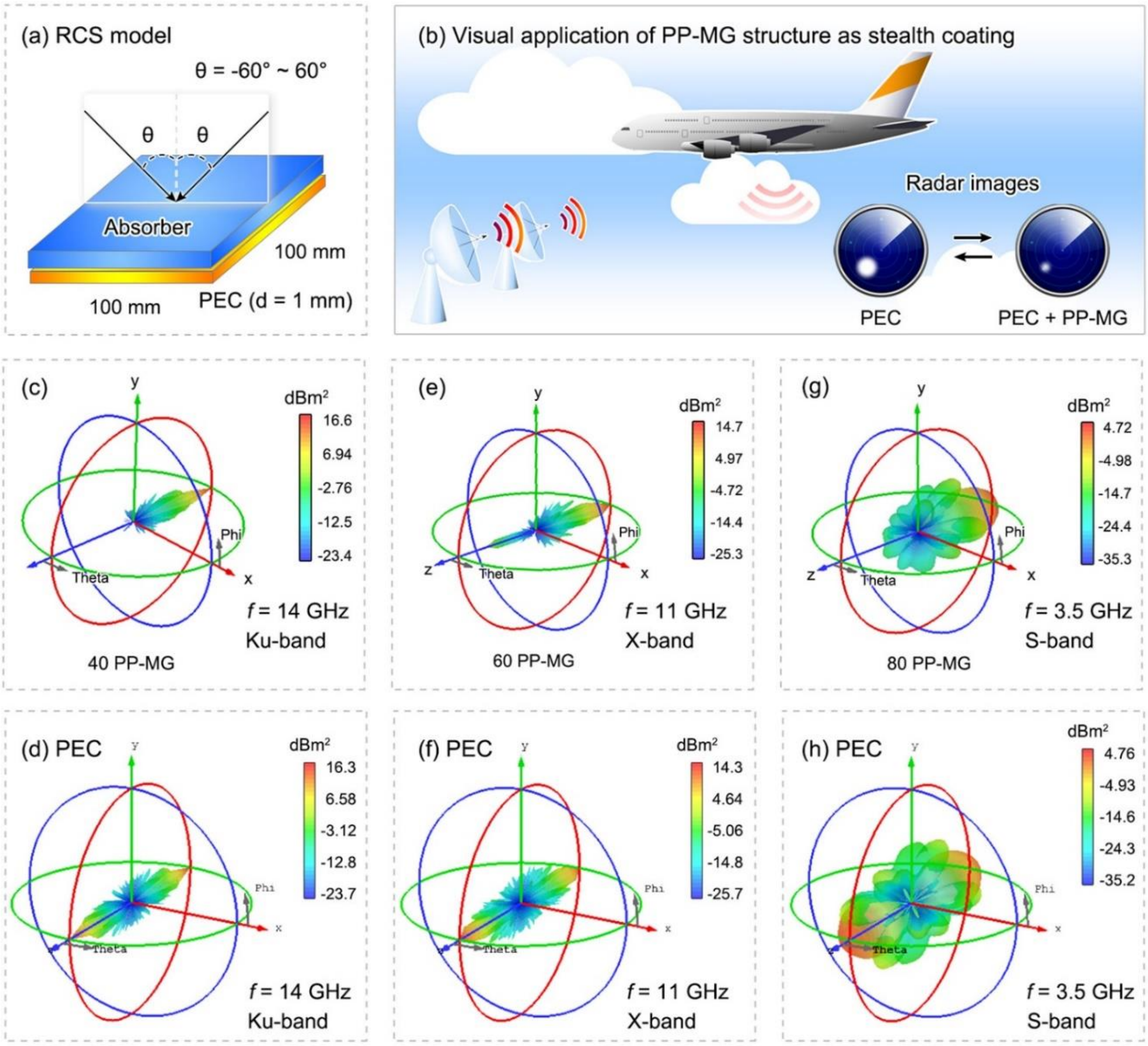

Figure S9. a) RCS simulation model. b) Visual application of PP-MG heterodimensional structure as stealth coating. 3D radar wave scattering signals of (c) 40 PP-MG and (d) PEC at 14 GHz (Ku-band). 3D radar wave scattering signals of (e) 60 PP-MG and (f) PEC at 11 GHz (X-band). 3D radar wave scattering signals of (g) 80 PP-MG and (h) PEC at 3.5 GHz (S-band).

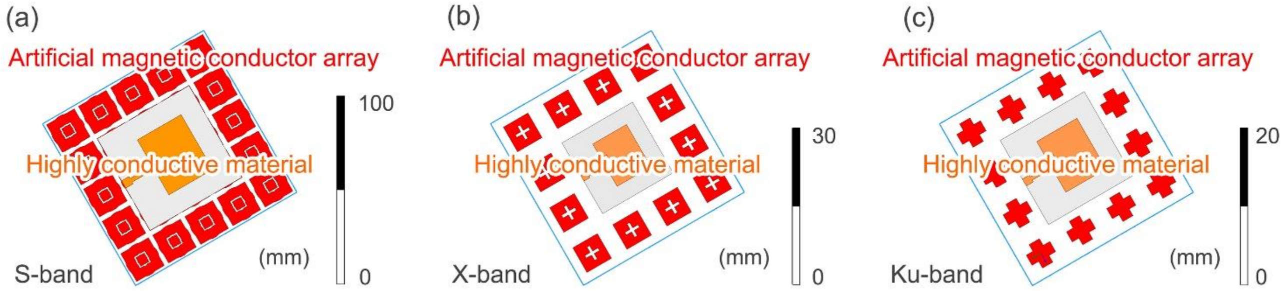

Figure S10. Geometries and dimensions of artificial magnetic conductor-backed antennas.

References

[1] X. X. Wang, M. Zhang, J. C. Shu, B. Wen, W. Q. Cao, M. S. Cao, *Carbon* **2021** *184*, 136.
